# Book Review

**Published:** 1897-03

**Authors:** 


**Boletin del Consejo Superior de Salubridad.** Mexico. Mayo 22 de 1896.—The medical men of the State and city of Mexico were determined not to let the centenary of the first successful vaccination by Edward Jenner pass without celebrating the event, and for this purpose they published a special number of their *Journal*, which contains a portrait of the doctor, two articles from the pens of Drs. J. R. de Arellano and Fernando Malanco, a poem composed for the occasion, and an official list of the number of persons vaccinated in the State since the lymph was introduced into Mexico by Dr. Balmis in 1804. After alluding to the fearful ravages of small-pox in different parts of the world anterior to 1796, the contributions proceed to describe with great accuracy the circumstances which led up to the immortal discovery which conferred untold blessings on future generations, and, especially, on the Mexicans themselves; for it appears from this pamphlet that a person in that climate who is once efficiently vaccinated will never in any event whatever contract small-pox, the protection for life being complete. Dr. de Arellano says: "One to whom the entire human race may look up with gratitude and respect, one who may with reason be called 'The great Benefactor of the Universe' is Edward Jenner, the grand discoverer of Vaccination." Further on he ejaculates: "May eternal praise and laudation be given to his sublime genius, and before him all generations should bow the knee!"

**Chemical Notes and Equations.** By G. H. GEMMELL. Pp. xi., 244. London: Baillière, Tindall & Cox. [1896].—This little book is very exactly described by its title, and is not unlike several others published under similar circumstances. It comprises the points generally touched upon in a course of elementary chemistry, and would form a convenient companion in the lecture room.

**Elementary Anatomy and Surgery for Nurses.** By W. McADAM ECCLES. Pp. xv., 158. London: The Scientific Press, Limited. 1896.—This is a book devised to meet the requirements of the scientific nursing fad. Into it are introduced chapters on anatomy, physiology, and surgery, somewhat curiously shuffled. Thus, the second chapter deals with the osseous system, whilst chapter III. is devoted to the process of inflammation and the healing of wounds, the idea presumably being to avoid tiring the mind with the effort which would be necessary to grasp the facts concerning the structure of the body if presented in unbroken series. It is evident that the author has experienced considerable difficulty in determining how much knowledge he ought to impart concerning structural details, and it is as clearly evident that he has not surmounted this difficulty. It may be confidently stated that the nurse who has carefully studied this book and committed its details to memory, will have a very curious smattering of knowledge. Drawings are introduced somewhat copiously. Those which have been executed specially for the book are exceedingly crude, and can serve but little practical purpose, for they are for the most part unprovided with reference letters. It would, for instance, be interesting to know what any nurse could make of Fig. 47. In Fig. 49 we are shown *two* posterior chambers of the eyeball. They can scarcely both be accurate. These are but a few examples of the character of this book. It exhibits a marked want of literary style, and is in some places deficient in ordinary grammar. A careful study of this work would result in the gain of a little knowledge by a nurse, but then a little knowledge has been said to be a dangerous thing. We have heard a nurse speak of pericarditis of the tibia! We leave the reader to imagine what she might say after the study of the smattering of facts grouped together here.

**Clinical Diagnosis.** By W. G. Aitchison Robertson, M.D. Pp. 366. London: The Scientific Press, Limited. 1896.—There are now many excellent books covering this field, and to establish a place for itself a new one must offer some special advantage. In his preface the author states that he has confined himself to those methods which he has found easy, rapid, and trustworthy. The book cannot, of course, take the place of the larger treatises on the subject as a work of reference. It is of convenient size, has a good index, and may thus be readily referred to for the ordinary tests used in clinical work. As to some of the more elaborate processes of research that are described in it, more detailed instructions are needed to make the descriptions adequate for successfully carrying them out. The book might either be increased in size, or, on the other hand, it might be still further cut down by leaving out all but the simple tests. The examination of the urine is more fully considered than the other subjects, taking up half the book: we notice that the total urinary solids are given differently in two places. The illustrations are not at all good, and the scale of magnification is not given.

**Aids to Medicine.** By Norman Dalton, M.D. Four Parts in two Volumes. Pp. vi., 125; vii., 128; vii., 132; vi., 122. London: Baillière, Tindall & Cox. [1896].—This professes to be a compendium of the practice of medicine in two small volumes, for assisting students in acquiring the minimum of knowledge necessary to pass the examination for the lower-grade qualifications. No doubt the book will fairly well meet such requirement, where a fuller work has also been used by the student habitually. We can not think, however, that Dr. Norman Dalton, Physician to King's College Hospital, will exalt his reputation by a publication of this description.

**Angio-neurosis: being Studies in Diseases of the Vaso-motor System.** By W. Ramsay Smith, M.B. Pp. 78. Bristol: John Wright & Co. [n.d.].—The author describes two kinds of skin disease, general erythema and papular erythema, and then proceeds to unite them together under the generic title of angio-neurosis. But not the slightest evidence is given that either of them is due to a neurosis, or even to vaso-motor disturbances. A conspicuous feature in the book is the amount of padding: for instance, we fail to see any connection between the treatment of typhoid fever by internal antiseptics and the skin diseases above mentioned. This book forcibly reminds us of the saying of Don Quixote, that "there are men who compose books and toss them out into the world like fritters."

**The Hydro-electric Methods in Medicine.** By W. S. Hedley, M.D. Second Edition. Pp. 115. London: H. K. Lewis. 1896.—The second edition of this work is a decided improvement on the former issue, inasmuch as it specially deals with the therapeutical application of hydro-electricity. The valuable but very technical information on the conversion of "current from the main" for therapeutical purposes is now published separately and is noticed below. The author gives an excellent and clear description of the hydro-electric bath in sufficient detail, so that practitioners with but a minimum amount of practical knowledge of the physics of electricity are enabled to utilise this valuable appliance. The sinusoidal current bath and allied forms, the electric vapour bath and the electric hot-air bath, the electric douche, and local hydro-electric applications are described in some detail. The value of the work would be further enhanced, and the volume made more interesting, if we were told more fully what these various methods do for our patients, instead of simply how they may be applied; nevertheless, the book is one which at present fills a gap, and affords welcome information on methods which have hitherto been confined to a very small circle of medical practitioners.

**Current from the Main.** By W. S. Hedley, M.D. Pp. 34. London: H. K. Lewis. 1896.—This booklet, treating of the medical employment of electric lighting currents, is based upon some articles by the author which appeared in the *Lancet;* but certain points, especially those dealing with the constructive details of apparatus, are here considered more fully. In the first part Dr. Hedley discusses with tolerable completeness the precautions necessary to avoid the passage through the patient of dangerously strong currents in using the electricity of public supply for medical purposes. The suggestions he makes are for the most part excellent, and indicate the practical character of his acquaintance with the subject. Why he should work out to five significant figures a resistance which depends on such uncertain quantities as baths and earths it is, however, difficult to see. The second part of the book is largely devoted to the discussion from the medical standpoint of the two kinds of supply—direct and alternating; the question of contact of the electrode with the skin being treated at some length. Here, too, most of what is said is good, though we cannot agree with the author's account on p. 15 of what happens on closing a circuit. In the case of a large E.M.F. applied to a resistance in series with the patient the current will attain its full value more rapidly than with a small E.M.F. and no external resistance, the inductance being the same for both; but it will not exceed its steady value without the addition of capacity at proper points on the circuit; nor is it likely to oscillate appreciably in such circuits as are here contemplated. The book concludes with a description of a neat "reducer of potential" devised by Mr. Quin. There is a slip on p. 4 in the expression for the resistance of two conductors in parallel. The fraction in line 3 should be inverted.

**The Modern Treatment of Stone in the Bladder by Litholapaxy.** By P. J. FREYER, M.D. Second Edition. Pp. 120. London: Baillière, Tindall and Cox. 1896.—Dr. Freyer has now performed Bigelow's operation of litholapaxy 610 times with only 11 deaths, so there is no wonder he speaks of it in terms of unstinted praise. It is to Keegan and Freyer that we owe the extension of this operation to children, and now most surgeons will agree with the author that "litholapaxy is no longer on its trial; it is now a firmly established practice; it has completely replaced lithotrity; and is destined to replace all forms of lithotomy, save in very exceptional cases." The present edition of this work gives us the latest modifications and improvements in the instruments employed in this operation, and, as might be supposed, considerable space is afforded to the operation as applied to male children. No less than 171 children have been operated on by the author alone, with only two deaths, thus showing the safety with which it can now be performed. We congratulate Dr. Freyer on the value of his work, and on the simplicity with which he writes.

**Aids to Obstetrics.** By SAMUEL NALL, M.B. Fifth Edition. Pp. 145. London: Baillière, Tindall & Cox. [1896].—This little book, if used in the way intended by its author, may be found useful for the refreshment of the memory by students preparing for their final examination in midwifery. If, however, it be used as a substitute for the larger text-books, it will produce an effect harmful, not only from the educational point of view, but from that of the question of success or failure in examination; for its lack of amplification is misleading, while it contains numerous inaccuracies in matters of detail. Used simply for mnemonic purposes in conjunction with a standard text-book it will be harmless, and possibly beneficial, although this is not certain, since a knowledge of practical points rather than mere book-work is what is most required. The "aids series" in general are of doubtful educational value, and this one is not exempt from their usual faults. Its limited use scarcely counterbalances the tendency to induce the student to look upon the memory of the descriptive words, apart from an intelligent mental picture of any particular process, as the object of his study of the literature of midwifery.

**Curettage of the Uterus: History, Indications, and Technique.** By J. W. BALLANTYNE, M.D. Pp. 36. Edinburgh: Oliver and Boyd. 1896.—We have here an interesting account of the history of the operation of curetting the uterine cavity since its introduction, with results not immediately satisfactory, by Rĕcamier. In the pamphlet are clearly set forth the conditions which call for its use, its possible dangers, advantages, and *technique*. Several illustrative cases are carefully described under the sections dealing with the conditions which may lead to the operation. The author is clear, and elaborates his subject with great detail: his remarks on *technique* are particularly noteworthy, and should correct a rather general impression that curetting is a simple and safe operation, of only slightly greater importance than passing a catheter. The pamphlet is very readable and instructive, without being dogmatic; the conclusions formed are sound and well weighed; debated points have the arguments for and against them stated clearly and without bias. It is difficult to see why it should be necessary to use a French word in describing a process which has an English, or at any rate Anglicised, name; in this respect German practice may be copied with advantage, although etymologically no doubt the author is correct.

**Handbook of Diseases of the Ear.** By URBAN PRITCHARD, M.D. Third Edition. Pp. xvi., 275. London: H. K. Lewis. 1896.—In preparing this third edition the author has largely re-written it, and has added about thirty pages of new matter, the result being that the book is somewhat larger, but is certainly considerably improved. It is written in a simple, easy style, without much of the minute technical detail found in many of the larger works, but containing all that is absolutely necessary for a good understanding of the nature and treatment of ear diseases. Among the new matter we notice especially a chapter devoted to the chief methods of operating for the removal of adenoid vegetations from the naso-pharynx. For this operation the author recommends the administration of a general anæsthetic, and the use of the finger-nail in preference to any other instrument. We do not agree with him in this preference; but we have no doubt that with constant practice the method may produce good results. The operation for removal of the ossicles in cases of chronic and intractable suppuration of the attic is favourably spoken of and well described. The curious condition known as Cholesteatoma is considered separately. A good elementary description of the intra-cranial complications met with in chronic middle ear suppuration is given, and an outline of the best methods of dealing with them: but we notice that in describing Ballance's operation for sinus thrombosis, the author tells us to tie the *external* jugular vein,—this is obviously a misprint for *internal*; but it might mislead the unwary, especially as in every other place where the operation is mentioned the vein to be ligatured is simply called "the jugular vein." Upon the whole we can recommend the book as one of the best small works on the subject, and as eminently suitable for students and practitioners.

**Du Traitement chirurgical de la Surdité et des Bourdonnements.** Par Dr. E. J. MOURE. Pp. 11. Bordeaux: Feret et Fils. 1896.—Dr. Moure here discusses the operation of removal of the ossicles, and particularly the operation of excision of the stapes for various forms of deafness, and comes to the sensible conclusion that while it is often useful in cases of caries with chronic suppuration, yet it should be recommended with great caution where it is done simply to relieve deafness and tinnitus.

**De la Prothèse appliquée au Traitement des Empyèmes de l'Antre d'Highmore.** Par Le Dr. SIMON DUNOGIER. Pp. 14. Bordeaux: G. Gounouilhou. 1896.—The author in this little pamphlet describes his method of treating suppuration in the maxillary antrum by the extraction of the first molar or second bicuspid tooth, and the insertion of a vulcanite plate having a pivot attached to its upper surface for keeping open the orifice, and an artificial tooth to its under surface to prevent deformity, and to keep food from entering the cavity during mastication. He directs that the apparatus should be removed daily, and the cavity syringed out. We consider that no better plan has yet been devised for treating this troublesome malady.

**The Anomalies and Evils of the Medical Administration of India.** By K. N. BAHADURJI, M.D. Pp. 70. London: A. Bonner. 1896.—This deals with a very old and long-standing question in India, which has been repeatedly discussed. The position of the medical profession in India is a strange and anomalous one. It has no parallel in any other country. It is the outcome of our occupation of the country since the days of Clive. We took India with the sword: we hold it still with the same weapon. There were no European medical men in India for many years, except those connected with the English army of occupation, the surgeons and assistant-surgeons of English regiments, and, as time moved on, the old East India Company's army supplied the only educated medical men; and these men were naturally appointed to Civil positions, and became the only practitioners in the country, first to their own countrymen, and gradually to the Native population. To-day there are three distinct services, two purely military—Her Majesty's Army Medical Department and the Indian Medical Department—and one Civil Medical Department. The two former are covenanted, the latter is uncovenanted. This pamphlet contains a number of arguments, principally emanating from Native medical men and from the Native press, calling loudly for reform, on the ground that all the best medical appointments in the country are divided between the two Military Departments, and the Civil Department is left out in the cold. The Civil Department is recruited principally from the natives of India, and they complain that they are overworked, and do not receive the same consideration as the other two. Their case is a hard one; but there is something to be said on the other side. If all the good appointments are taken from the two Military Departments and handed over to the Civil Branch, the one inducement to highly educated medical men to enter either of the Services will be removed.

**Disease and Defective House Sanitation.** By W. H. Corfield, M.D. Pp. 55. London: H. K. Lewis. 1896.—This small volume reproduces the substance of two of the Harveian Lectures delivered by Dr. Corfield in December, 1893. It gives in readable form, with the aid of suitable diagrams, the chief points of importance connecting disease and defective sanitary arrangements. The wide diffusion of accurate knowledge on these points is both useful and necessary.

**Food in Health and Disease.** By I. Burney Yeo, M.D. New and revised Edition. Pp. viii., 592. London: Cassell and Company, Limited. 1896.—This new edition of a well-known and valued text-book has been enlarged and improved. That it has been a practically useful guide to the study of dietetics has been shown by the need for many reprints since the publication of the first edition in 1889. The consideration of the adaptation of scientific dietetics to the needs of the sufferer from acute and chronic diseases forms the most useful part of the volume. It is one of the best of guides on all points relating to the feeding of the sick. It is a much better looking book than the first edition, which we reviewed soon after its appearance.

**Dress and Health: an Appeal to Antiquity and Common Sense.** By Charles Moore Jessop. Pp. 47. London: Elliot Stock. 1896.—These two essays, read before the British Medical Association in 1887 and 1889, are worthy of reproduction, although there is less need for them now than formerly. The author was quite a pioneer in protesting against the deluging of patients with meat washings and the great waste of meat by disposing of the fibre as refuse: his principle is that there should be neither refuse nor remainder. He finds that four ounces of meat can be suspended in one pint of water, and that two to four ounces of such fluid given every three or four hours are sufficient for any sick man.

**Dr. Tiebel's Official Handbook of Spas, Watering Places, and Health Resorts.** Pp. vii., 74. London: Charles Letts & Co. [1896].—This is a useful little *multum in parvo*, a curious mixture of notices of spas with advertisements of pills, cancer serum, and German rusks. The only mention of Bournemouth is an advertisement of a boarding-house. Bath and Droitwich have each a page, Harrogate has four, but they all look like advertisements. The book is not one for serious criticism, nor is it worthy of much commendation. We wonder in what sense it deserves the designation "Official."

**The Johns Hopkins Hospital Reports.** Vol. V. Baltimore: The Johns Hopkins Press. 1895.—This volume of these valuable Reports begins with an elaborate and painstaking paper by Dr. W. S. Thayer and Dr. J. Hewetson upon "The Malarial Fevers of Baltimore." No less than 616 cases have been under their observation, and in 544 cases the malarial organism was discovered and studied. In Baltimore there are three forms of malarial fever—the tertian, quartan, and æstivo-autumnal. The organism found differed in these three fevers. Careful descriptions are given of the development of the intracellular and extracellular forms of these parasites. Comments are made upon the nature of crescentic bodies and flagella, but these observers are not able to come to any satisfactory conclusion as to their nature, and consider that they require further study. A consideration of some fatal cases of malaria by Dr. L. F. Barker follows. In one of these cases there were areas of necrosis in the liver, surrounded, as is usually the case with such patches, by leucocytal infiltration. Dr. Barker seems to think that such a condition is probably the first step towards the cirrhosis of this organ, which has from time to time been described as a sequence of malaria. This writer makes some interesting remarks upon phagocytosis in malaria, and gives an illustration showing macrophages in the spleen with malarial parasites in their interior. Valuable though these papers upon malaria are, the majority of physicians will probably be more interested in the articles upon typhoid fever. Dr. Osler gives a further analysis of 160 cases, making altogether 389 cases studied by him. The varied characters of the rash, the temperature, severity of diarrhœa, frequency of hemorrhage, perforation, and other rare complications, as illustrated by these cases, are passed under review. Amongst other interesting points are the records of two cases of apparent recovery from perforation. A short paper upon "The Cold-bath Treatment of Typhoid Fever" follows. In 299 cases there was a mortality of 6.6 per cent., which, as Dr. Osler points out, is very favourable compared with the statistics of cases treated by other methods. For example, the Report of the London Fever Hospitals, issued in 1893, gave a mortality of 17 per cent. for typhoid fever. Other valuable papers follow. One by Dr. Blumer on "Pyuria in Typhoid Fever," in which the bacillus coli communis was the most common organism found, would lead us to suppose that serious secondary lesions may generally be due to other organisms than the typhoid bacilli. Other papers, however, by Dr. Flexner on various secondary infections, and by Dr. Harold Parsons on post-typhoid bone lesions, show that the typhoid bacillus may be responsible for a great deal more than the production of intestinal ulceration. To refer again to the pyuria described by Dr. Blumer, it may be said that it was found as often as ten times in sixty cases. This volume closes with three other papers by Dr. Osler and one by Dr. Reed. The papers by Dr. Osler are upon "Neuritis during and after Typhoid Fever," "Chills in Typhoid Fever," and "A Study of the Fatal Cases." The paper by Dr. Reed is upon "The So-called Lymphoid Nodules of the Liver in Typhoid Fever." We cannot close this brief review without expressing our admiration for the continuous stream of able work that issues from the institution which gives these Reports their name.

**Edinburgh Hospital Reports.** Volume IV. Edinburgh: Young J. Pentland. 1896.—These Reports contain, as the previous volumes led us to expect, a large number of papers—forty-nine in all—of great interest and value. They afford a striking testimony to the value and extent of the scientific work on the investigation of disease in its clinical and pathological aspects done at the Edinburgh Royal Infirmary. We cannot attempt to give an account of so many papers here, but can only say that these Reports deserve to be widely read. They are as usual well printed, and many of the papers are beautifully illustrated. The book does great credit to everyone concerned in its production.

**Thirty-fifth Annual Report of the Cincinnati Hospital.** Cincinnati: The Commercial Gazette Job Print. 1896.—The cases reported in this volume are hardly given in sufficient detail to be of value. The numerous tables of cases treated in the Hospital may be of use to the statistician, but will not do much to advance medical science.

**Transactions of the College of Physicians of Philadelphia.** Third Series. Vol. XVI. Philadelphia: Printed for the College. 1894.—A remarkable paper on "Hydrophobia in the United States" tends to the conclusion that there is nothing specific in the saliva of a so-called mad dog, and that hydrophobia is not a specific inoculable disease. It is quite refreshing to find a writer so courageous as this: he gives altogether a new aspect to the hydrophobia question. Various papers on acute and chronic appendicitis, and Dr. Stewart's researches on the reactions of nucleo-albumin (erroneously styled mucin) with the commonly employed urinary albumen tests are the most noteworthy features of this volume.

**Transactions of the Grant College Medical Society, Bombay.** From January to December, 1895. Bombay: The "Tatva-Vivechaka" Press. 1896.—This volume is of more than local interest, and shows that our Indian *confrères* are well versed in the latest developments of modern science. The book stands in need of a Table of Contents and an Index. A more careful correction of the proofs would prevent many of the elementary errors which we notice on almost every page.

**Transactions of the British Balneological and Climatological Society.** 1896.—**The Journal of Balneology and Climatology.** Vol. I. Part I. January, 1897. London: John Bale & Sons.—The Society with the massive title given above has come to the conclusion that, in addition to records of its proceedings, a quarterly journal, dealing with the subjects which it is its purpose to consider, is necessary for the world's enlightenment. The editor of the journal is evidently impressed with the magnitude of the task before him, and he will certainly have to exercise considerable care and discretion in accepting papers, in order to avoid the appearance in his pages of that which might be considered to be little more than advertisement of certain health resorts. There should be no undue laudation of these. In the journal there is a paper on the climate of Ilfracombe. We in this neighbourhood well know how admirably adapted it is for many patients; but no scientific purpose can be served by a writer who attempts to class it with "the Riveira *(sic)*, South Africa or Australia." The most interesting article in the journal is Dr. Ewart's on the decrease of ague and aguish affections in London. In future numbers the reports of the meetings should be considerably shortened. The Society evidently has some intention of being practical, as it devotes over fifteen pages of the *Transactions* to a discussion on the need of increasing travelling facilities in connection with British health resorts. By the rules of the Society homœopaths and persons carrying on "irregular practice"—whatever that may mean—and women are excluded. This is unfair on legally-qualified women who are as much members of the profession as men are, and does not confirm the statement that the rules "have been framed upon the broadest and fairest principles."

**Transactions of the Obstetrical Society of London.** Vol. XXXVII. London: Longmans, Green, and Co. 1896.—Space allows us to only briefly call attention to some of the communications which make up this very interesting and useful volume. In "Observations on the Temperature, Pulse, and Respiration during Labour and the Lying-in," Dr. Probyn-Williams and

Mr. Lennard Cutler record some valuable evidence of the variations from the normal caused by those conditions. As regards the pulse, they consider that the low rate of pulse usually described as obtaining soon after delivery is exaggerated, but that a diminution of rapidity does take place. Their observations on the temperature agree with those of Dr. Giles noticed in last year's *Transactions*. Respiration rate follows the pulse rate rather than the temperature. In a paper "On the Common Form of 'White Leg' after Confinement," Dr. Hubert Roberts gives details of sixteen cases. He classifies the condition, after excluding those due to pressure, certain general diseases, and general septic conditions, into (*a*) Cases associated with thrombosis apart from sepsis. (*b*) Cases of sepsis and thrombosis combined. He shows that the cases most commonly met with are those in group (*a*), while true phlegmasia alba dolens, placed under group (*b*), is comparatively infrequent. Drs. T. G. Stevens and W. S. A. Griffith contribute some valuable "Notes on the Variation in Height of the Fundus Uteri above the Symphysis during the Puerperium." The practical importance of these observations is, that any serious deviation from the normal rate of fall should act as a danger signal (provided that certain sources of error which are mentioned are excluded) and lead to the suspicion of retained clot, etc., and the possibility of sapræmia, while the getting-up of the patient should not, as is often the case, be governed by rule of thumb, but by the actual disappearance of the fundus on its becoming once more a pelvic instead of an abdominal organ. Dr. G. D. Robinson, writing "On Certain Micro-organisms of Obstetrical and Gynæcological Interest," points out the constant presence of streptococcus pyogenes in the blood and tissues in fatal cases of puerperal sepsis. Its importance is due to the fact that it can penetrate the deeper layers of uterine tissue, instead of being filtered off by the mucous membrane, as are others. The bacillus coli communis is also mentioned, and its importance as the possible cause of puerperal complications demonstrated. To this cause have been attributed cases in which high fever, rigors, etc., in puerperal women have occurred with constipation, and disappeared on removal of the constipation; and undoubtedly peritonitis might occur in cases where injury to the rectal wall has occurred in cases of neglected protracted labour, with the head above the brim. The gonococcus is also shown to be a source of danger, but in a less degree, as it is said to die at the maximum temperature of even a hot day in this country.

**Transactions of the American Dermatological Association.** New York: Geo. L. Goodman & Co. 1896.—We have here a volume of 195 pages, with many illustrations, some of which are coloured, and which are, upon the whole, well done. The first plate, illustrating Dr. Fordyce's case of angiokeratoma of the scrotum, is the best one of this disease we have ever seen.

The statistical returns of this Association are of very great interest, and embrace 24,321 cases. Of them eczema forms 25.78 per cent.; psoriasis about 2.5 per cent.; dermato-syphilis rather over 10 per cent.; and herpes zoster 1 per cent. The returns for eczema are massed under one head only, although the table provides for seven subdivisions, exclusive of the seborrhœic form. They should either be classified or the distinction abandoned. The volume contains many interesting and useful papers, of which perhaps the most valuable is that on "Sleep in its Relations to Diseases of the Skin," by Dr. Duncan Bulkley, of New York. He carefully discussed the causes of sleep-disturbance under six classes; viz., digestive, toxic, circulatory, nervous, psychic, and cutaneous. He deprecated the use internally of opiates, and especially when the cutaneous surfaces were involved. Antipyrin, in repeated doses of five grains, seems in his hands to have relieved osteocopic and neuralgic pains in syphilis, and also the neuralgia following herpes zoster in old people. We should prefer antipyrin, and we cannot overlook the comfort obtained by opiates in old syphilitic subjects. We are sorry to find such an excellent volume sent out with neither a table of contents nor an index.

**The Year-Book of Treatment for 1897.** London: Cassell and Company, Limited.—This annual, while needing no introduction to practitioners, may once more be assured a welcome, for it fully maintains the reputation of previous issues. The staff of contributors has been changed in respect to some of the sections. Diseases of the lungs are now looked after by Dr. Gustave Schorstein; diseases of the kidneys by Dr. F. Boyd; rhinology and laryngology by Dr. St. Clair Thomson; hygiene by Dr. Willoughby; therapeutics by Dr. Nestor Tirard. These names are a guarantee of able work.

**The Medical Annual.** Bristol: John Wright & Co. 1897.—We think that the value of this Annual would be enhanced if, in dealing with various methods of treatment and the exhibition of new remedies, the authors of the articles would separate the wheat from the chaff. To do this implies wide clinical experience, such as may not be possessed by every one who is qualified to act as an abstractor of the statements of various writers. What the busy practitioner wants in such a book as this is to be able to turn to a certain disease, and to find accurate guidance given to him in the selection of trustworthy new treatment. The quality of the articles in this volume varies greatly, as is inevitable where the staff of contributors is so large, and one of them, that on post-nasal adenoids, is distinctly misleading and retrograde. We hope that no one will deny efficient relief to children suffering from this serious trouble, because he reads this article and finds opinions expressed there which are at variance with all clinical experience. The benefits of thorough removal of post-nasal adenoids are incontestable. The book is very well got up, the illustrations are numerous and good, and it is well worthy of the attention of the general practitioner.

**The Scottish Medical and Surgical Journal.** Edinburgh: William F. Clay.—This new monthly Journal, the first number of which appeared in January, claims to be a national one, and to represent medical work done all over Scotland. Edited by Dr. William Russell and published under the auspices of a powerful staff in Aberdeen, Dundee, Edinburgh, and Glasgow, it is under the sole direction of members of the profession whose names and positions are a guarantee of its representative character and of the quality of the work likely to be found in its pages. The first number contains eight original papers, together with the usual reviews and notices of books, concise reports of meetings of medical societies, a digest of recent work and literature, and Scottish medical news. We may venture to predict that our new contemporary will prove a brilliant success.

**The Middlesex Hospital Journal.** Vol. I. No. I. January, 1897. London: Mitchell and Hughes.—The first number of this journal contains an excellent characteristic portrait and short biography of the late J. W. Hulke. There is a practical illustrated paper by Mr. Henry Morris on stone in the bladder, dealing with perineal lithotrity and the relations of the X rays to diagnosis; notes on the condition of the knee-jerks in fracture and dislocation of the spine, by Dr. Campbell Thomson; notes on a case of transposition of viscera; a history of the Hospital Medical Society, by Dr. Wynter; and much local information. With such a good start, the journal, which is to appear five times in the year, promises well.

**Professional Nurses' Diary, 1897.** London: Burroughs, Wellcome & Co.—This time last year we spoke in high terms of praise of this excellent production. This year's issue surpasses its predecessor in outward attractiveness, and is at least equal to it in general usefulness.

That excellent periodical the *Journal of Cutaneous and Genito-Urinary Diseases* is now edited by Drs. Jas. C. Johnston and George K. Swinburne, and issued by the The Physicians Publishing Co., 115 West 84th Street, New York.

Even the old-established *Edinburgh Medical Journal* has undergone a change. The editor is now Dr. G. A. Gibson, who does not rely entirely upon his Scottish brethren for his material, but is largely helped by London. The Journal is now published by Mr. Young J. Pentland, a guarantee that its typographical and pictorial attractiveness will be of the highest character.

The editors of *Mathews' Medical Quarterly* announce that, beginning with this year, the publication is to be known as *Mathews' Quarterly Journal of Rectal and Gastro-Intestinal Diseases.*

